# Emergence of concurrently transmissible *mcr-9* and carbapenemase genes in bloodborne colistin-resistant *Enterobacter cloacae* complex isolated from ICU patients in Kolkata, India

**DOI:** 10.1128/spectrum.01542-24

**Published:** 2025-02-06

**Authors:** Gourab Halder, Bhaskar Narayan Chaudhury, Priyanka Denny, Mandira Chakraborty, Subhranshu Mandal, Shanta Dutta

**Affiliations:** 1 Division of Bacteriology, ICMR-National Institute for Research in Bacterial Infections formerly ICMR-NICED, Kolkata, West Bengal, India; 2 Division of Microbiology, Peerless Hospitex Hospital, Kolkata, West Bengal, India; 3 Collaborative Research Center for Infectious Diseases in India, Okayama University, JICA Building, ICMR-NIRBI, Kolkata, West Bengal, India; 4 Division of Microbiology, Calcutta Medical College, Kolkata, West Bengal, India; 5 Division of Microbiology, CNCI, Rajarhat, Kolkata, West Bengal, India; JMI Laboratories, North Liberty, Iowa, USA

**Keywords:** *mcr*-9, colistin resistance, CR-ECC, MLST, WGS, efflux pump, PFGE

## Abstract

Colistin resistance in carbapenem-resistant *Enterobacter cloacae* complex (CR-ECC) infections has grown expeditiously but detecting the underlying mechanism of resistance is often challenging in clinical settings. This study, first of its kind from India, determined the resistance mechanisms and characterized colistin-resistant hospital isolates. Twenty-nine bloodborne CR-ECC isolated from ICU patients of eight tertiary care hospitals in Kolkata, India between 2022 and 2023 were screened for colistin resistance. The plasmid-encoded *mcr*-9 gene, *acrAB-tolC* efflux pump (EP) & *phoP/Q,* and *pmr A/B* two-component system (TCS) involved in colistin resistance were examined. In addition, AMR gene profiling and molecular subtypes of *mcr*-9-producing CR-ECC isolates were also investigated. All study isolates showed resistance to ≥5 antimicrobial classes and six (21%) of them were colistin-resistant. The *mcr*-9 gene transferable by IncHI2-HI2A plasmid was detected in both colistin-resistant (67%) and colistin-sensitive (4%) CR-ECC isolates. The *bla*NDM-5 gene was significantly (*P* < 0.05) associated with isolates co-harboring *mcr*-9 genes. A ≥8-fold increase in minimum inhibitory concentration (MIC)^colistin^ was observed in the *mcr*-9-producing colistin-sensitive strain after induction. Overexpression of *acrA, ramA, soxS*, *phoP/Q,* and *pmrA/B* genes was found in non-*mcr*-9-producing colistin-resistant isolates. The resistance to colistin in the wild-type appeared to be mediated in part by the *mcr*-9 gene, an active EP, and regulatory TCS. The *mcr*-9-producing isolates were typed into ST932, ST270, and ST1997 by MLST. Heterogeneity (29 pulsotypes; 48.40% similarity coefficient) among the circulating CR-ECC isolates was revealed by PFGE. Robust monitoring of *mcr* genes in both colistin-resistant and -sensitive strains is warranted to curb the menace of AMR in nosocomial pathogens.

**IMPORTANCE:**

Carbapenem-resistant *Enterobacter cloacae* complex (CR-ECC) has become a global nosocomial pathogen in last few years. Colistin, the “last resort antibiotic,” is being widely used in the treatment of CR-ECC and, consequently, there has been a brisk rise in colistin-resistant CR-ECC isolates. This study was necessitated by the dearth of a comprehensive molecular investigation of colistin-resistant CR-ECC from India. The notorious IncHI2-HI2A plasmid-borne mcr-9 gene along with active acrAB-tolC efflux pump and phoP/Q-pmr A/B two-component system was found to mediate colistin resistance in the study isolates. Interestingly, the mcr-9 gene was also discovered in colistin-sensitive strains and MIC of colistin was found to increase under colistin pressure. Diverse phylogenetic clones along with novel sequence types were detected. This study highlights the necessity for intense monitoring of mcr-9 in conjunction with the existing epidemic clones of CR-ECC strains harboring diverse arrays of transmissible AMR genes.

## INTRODUCTION

*Enterobacter* spp., an opportunistic pathogen and integral member of the “ESKAPE” consortium (*Enterococcus faecium*, *Staphylococcus aureus*, *Klebsiella pneumoniae*, *Acinetobacter baumannii*, *Pseudomonas aeruginosa*, and *Enterobacter* spp.), has emerged as a major etiological agent of nosocomial infections like pneumonia, urinary tract and gastrointestinal tract infections, meningitis, and septicemia globally (1). Of the 19 species of Enterobacter genus (2), the *Enterobacter cloacae* complex (ECC), comprising *E. cloacae, E. hormaechei, E. kobei, E. asburiae, E. ludwigii, E. roggenkampii*, and *E. mori* are primarily human pathogens (2), and *E. cloacae* alone is known to cause 65%–75% of all Enterobacter infections, including hospital-acquired infections (3). Among the *Enterobacteriaceae* family, it ranks third in India as an underlying cause of bloodstream infections (BSIs) (4).

ECC is usually synonymous with multidrug resistance (MDR) phenotype primarily attributable to its intrinsic resistance and remarkable ability to acquire mobile genetic elements (MGEs) harboring multiple resistance genes. The chromosomally encoded AmpC- type β-lactamase makes it intrinsically resistant to numerous antibiotics, including first- and second-generation cephalosporins, cephamycin, ampicillin, and amoxicillin/clavulanic acid (5). The new-generation drugs like aminoglycosides and fluoroquinolones, and third-generation cephalosporin has been rendered ineffective and gradually supplanted by the Carbapenem group (meropenem, imipenem, ertapenem, and doripenem) which is considered as “last-resort antibiotics,” in the treatment of multidrug-resistant ECC (MDR-ECC).

However, over the past decade, carbapenem resistance in ECC has increased rapidly, attracting clinical attention. The carbapenem resistance in ECC can be explained by various factors, including the presence of enzymes that hydrolyze carbapenems, activation of efflux systems, loss of porin, and hyperproduction of the chromosomally encoded cephalosporinase AmpC (1). Consequently, the advent of carbapenem-resistant ECC (CR-ECC) has posed major challenges to the treatment of infections in clinical settings.

Due to the MDR crisis and scarcity of new antibiotics, polymyxins (such as colistin or polymyxin B), a polycationic antimicrobial peptide considered critically important by WHO has been prescribed globally; but owing to its associated nephrotoxicity and efficacy concerns, it has been restricted as last line of treatment (6, 7).

Colistin resistance in ECC may be chromosomally mediated or by plasmid acquisition. While most of the studies have suggested that chromosomal mutations in two-component systems *phoP/Q* and/or *pmrA/B* and/or *mgrB* (regulatory gene) as well as the activation of two-component regulatory systems (*phoP/Q* and *pmrA/B*) triggered by environmental stress such as cationic antimicrobial peptides (CAMPs), low magnesium levels, or acidic pH leads to upregulation of *eptA* and *arn* operons which, in turn, confers lipopolysaccharide (LPS) modification by phosphoethanolamine or 4-amino-4-arabinose resulting in colistin resistance in ECC (8–11). However, these mutations in ECC are more diverse and unstable compared to *Klebsiella* spp. and *E. coli* (6). However, contradictory results were shown in a few studies (11). One study suggested that pmrAB may not have a role in colistin resistance in *E. cloacae* (11). Another study reported that neither *phoPQ* nor *pmrAB* was overexpressed in colistin-resistant *E. cloacae* isolates. Furthermore, the small membrane protein gene *ecr* (12), the inner membrane protein DedA (12), and the efflux pump *acrAB-tolC* (13) have been documented to impart colistin resistance in ECC.

In addition, plasmid-mediated colistin resistance genes (*mcr*) have contributed significantly to the evolution of colistin resistance in *Enterobacter* spp., which, in turn, has had a major impact on the efficacy and efficiency of colistin in treating infections caused by CR-ECC isolates. The initial *mcr-1* gene was identified in ECC in late 2015, and since then, nine homologous *mcr* genes (*mcr2-10*) have been found globally (14, 15), with *mcr-9* and *mcr-10* being more common in ECC (6). However, the exact frequency of *mcr-9/10* genes in ECC remains unknown (6).

Furthermore, the first isolated strain (*Salmonella* spp.) found to possess *mcr-9* was susceptible to colistin (16); however, subsequent laboratory cloning and overexpression of *mcr-9* led to colistin resistance (16). While some researchers have found no correlation between *mcr-9* expression and colistin resistance (17); there are others who have shown that *mcr-9* can stimulate colistin resistance when the gene is located upstream of *qseB-C* two-component system in ECC (18). In light of these findings, it remains unclear when *mcr-9* leads to an increased MIC for colistin; this may be due to variations in the host’s genetic makeup or other as-yet-unidentified regulatory genes (19). Currently, an expediential increase in the number of reports of ECC isolates co-harboring *mcr-9* and carbapenemases, genes have been observed globally due to the rapid dissemination of self-transmissible IncHI2-IncHI2A plasmids (20).

Although reports of colistin resistance in ECC have been documented from several countries around the world (21–24), there is no data from the Indian subcontinent. Hence, this study was conducted to give insight into the emergence of colistin resistance in CR-ECC blood isolates from Kolkata over a 2-year period 2022–2023, as well as their AMR-gene profile and along with their molecular subtypes by different molecular subtyping methods. Furthermore, the genetic background of *mcr-9* harboring plasmids was investigated by whole-genome sequencing (WGS). In addition, a conjugation assay was conducted to explore the transmission dynamics of *mcr-9* and carbapenemase genes. We also studied the consequences of sub-inhibitory concentrations of colistin on the phenotype of CR-ECC strains carrying *mcr-9*. To the best of our knowledge, this is the first comprehensive analysis of the genetic background and spread of the *mcr-9* gene among CR-ECC isolates in India.

## MATERIALS AND METHODS

### Clinical study isolates

Bloodborne ECC isolates with carbapenem resistance were prospectively collected from central line blood of ICU patients of eight different tertiary care hospitals in Kolkata during the period of 2022–2023. Patients who had not received any carbapenem, colistin, or polymyxin B antibiotic prior to hospital admission were chosen for this study. Only the pure cultures of the CR-ECC isolates were sent to the bacteriology division at ICMR-NIRBI after they had been identified at the respective hospitals using the VITEK MS and VITEK 2 Compact system (bioMérieux, France). The isolates were reconfirmed by MALDI-TOF-MS (bioMérieux, France) at ICMR-NIRBI; and species-specific multiplex PCR helped in ECC species identification (25). This study was reviewed by the Institutional Board of Ethics Committee of ICMR-NIRBI.

### Antibiotic susceptibility testing and determination of MIC

Antibiotic susceptibility testing (AST) was performed by Kirby-Bauer disk diffusion method (26) using 22 different antibiotic discs (BD Diagnostics, USA) namely ampicillin (10 µg), amoxicillin-clavulanate (20/10 µg), ampicillin-sulbactam (100/10 µg), piperacillin-tazobactam (100/10 µg), chloramphenicol (30 µg), tetracycline (30 µg), trimethoprim-sulfamethoxazole (1.25/23.75 µg), cefepime (30 µg), cefotaxime (30 µg), ceftazidime (30 µg), aztreonam (30 µg), imipenem (10 µg), meropenem (10 µg), doripenem (10 µg), ertapenem (10 µg), ciprofloxacin (5 µg), levofloxacin (5 µg), amikacin (30 µg), gentamicin (10 µg), ceftazidime-avibactam (30/20 µg), minocycline (30 µg), and doxycline (30 µg). The broth microdilution method (BMD) was performed (27) to determine the MIC of the aforesaid antibiotics as well as those of colistin, ceftaroline, and tigecycline (Sigma Aldrich, USA). The results of AST and MIC were interpreted following the Clinical and Laboratory Standards Institute (CLSI) guideline (CLSI, 2022) (28) and (29, 29) using *E. coli* ATCC25922 strain and *Pseudomonas aeruginosa* ATCC 27853 as the control strain. MIC_50_ and MIC_90_ were estimated as per standard protocol (30).

### Determination of AMR genes

The presence of colistin-resistant genes (*mcr*-1 to *mcr*-10) (31), carbapenemases (*bla*_NDM_, *bla*_OXA-48_ like, *bla*_KPC_, *bla*_VIM_, *bla*_IMP_, *bla*_SPM-1_, *bla*_GIM-1_, *bla*_SIM-1_, *bla*_SME_, *bla*_GES_), Extended spectrum β-lactamases (ESBL) (*bla*_CTX-M_, *bla*_SHV_, *bla*_OXA_, *bla*_TEM_); AmpCs (*bla*_ACC_, *bla*_FOX_, *bla*_MIR/ACT_, *bla*_CMY_, *bla*_DHA_) (32), 16S rRNA methylase-encoding genes (*armA*, *rmtA*, *rmtB*, *rmtC*, *rmtD*) (33), and plasmid-mediated quinolone resistance (PMQR) genes (*aac(6*′*)-Ib-cr, qepA, qnrA, qnrB, qnrC, qnrD, qnrS*) (34) were verified by PCR followed by Sanger sequencing. The sequences obtained were then entered in the Basic Local Alignment Search Tool (BLAST) algorithm of NCBI to confirm the respective alleles.

### Conjugation assays

We performed a liquid mating assay to study the conjugal transferability of AMR genes in study strains (CR-ECC carrying *mcr-9* positive) using plasmid-free, sodium azide-resistant *E. coli* J53 strain and plasmid-free ampicillin-resistant clinical *E. coli, K. pneumoniae, S.* Typhi isolates (Table S1) as recipients. Concisely, pure colonies of both the donor and recipient cells from Chrome Agar plates (Difco) were inoculated into 3 mL of LB broth and incubated for 18 hours at 37°C in a shaker incubator. Subsequently, the donor and recipient were mixed in fresh LB broth in 1:1, 1:5, and 1:10 ratios, respectively, and incubated at 37℃ without shaking. After 18 hours of incubation, the mixture was serially diluted and spread on chrome agar plates containing 100 mg/L of sodium azide or 16–32 mg/L ampicillin (depending upon the recipient) and varying concentrations of meropenem (ranging from 1 mg/L to 3 mg/L) or colistin (0.5mg/L to 2 mg/L) (Sigma-Aldrich, St. Louis, MO, USA) and incubated. Standard biochemical tests followed by VITEK 2 confirmed the transconjugants (TC). The BMD method was used to determine the MIC of carbapenems (meropenem, doripenem, and ertapenem) and colistin in TC, while PCR (using the plasmid DNA of the TC as a template) was carried out using the aforementioned primers to determine the transfer of AMR determinants.

### Determination of mechanisms of colistin resistance

#### Role of efflux pumps by efflux pump inhibitor

The efflux pump’s role in colistin resistance was phenotypically determined by comparing the MICs of colistin both in the absence and presence of the efflux pump inhibitor (EPI)-carbonyl cyanide 3-chlorophenylhydrazone (CCCP; 10 mg/L) (Sigma-Aldrich) (35). Separate experiments were carried out in triplicate to determine MIC using the BMD method. A MIC reduction of four times or more in the presence of EPI is considered significant (24).

#### Role of *mcr*-9 gene by colistin induction assays and determination of MIC of the colistin-induced strain

The colistin induction assays were carried out following a modified version as described previously (18, 36). In short, *mcr-9* carrying colistin-resistant CR-ECC and *mcr-9* carrying colistin-sensitive CR-ECC were inoculated separately into 5 mL LB broths. Following overnight incubation at 37°C with shaking, the bacterial culture was transferred to fresh LB broth supplemented with varying concentrations of colistin (1 µg/mL, 2 µg/mL, and 4 µg/mL) and incubated until the beginning of the log phase. Subsequently, mRNA extraction and RT-PCR were conducted. *E. cloacae* ATCC 13047 (colistin MIC: 0.125 µg/mL) and *E. hormaechei* ATCC 49163 (colistin MIC: 0.5 µg/mL) served as positive controls (without colistin stress).

Following induction, the MIC of colistin in colistin-induced strains was evaluated under normal conditions (without antibiotic stress) at different time intervals (day 1, day 7, and day 15) to check the change in MIC and whether there is any reversal in MIC. The broth dilution for MIC determination was done in triplicates.

### Real-time PCR for efflux pump (*acrA-B, tolC*) and two-component systems (*phoP/Q*; *pmrA/B*), *arnA*, *mcr*-9, and *qseBC* expression

The bacterial isolates were grown at 37°C in LB broth with and without antibiotic (colistin) stress. RNA was extracted from both cultures in the log phase using Trizol Reagent. Quantification and quality assessment of the extracted RNA was done by NanoDrop biophotometer plus (Eppendorf, Germany). The total RNA was digested with RNase-free DNaseI (New England Biolabs, USA) to avoid genomic DNA contamination. Subsequently, complementary DNA (cDNA) was generated using the QuantiNova cDNA Synthesis Kit (Qiagen) with DNaseI-treated RNA as the template. The expression level of genes efflux pump and its regulators (*acrA*, *acrB*, *tolC*, *ramA*, *soxS*, *soxR*), *mcr9* gene and its regulators *qseB* and *qseC* , two-component system (*phoP*, *phoQ*, *pmrB*, *pmrA*), *pmrC* and *arnA* gene using gene-specific primers (18, 19, 36) was estimated by real-time reverse transcription-PCR (RT- PCR) on Step One Plus Real-Time PCR System (Applied Biosystems). The 2^−ΔΔC^T method (37) was employed to determine the relative RNA expression levels by normalizing them with the ribosomal housekeeping gene *rpoB*. Three independent triplicate experiments were performed using the *rpoB* gene as an internal control (38). The relative expression of each target gene was calibrated by comparing it to the expression level (set as 1) of pan-susceptible *E. cloacae* ATCC13047 or *E. hormaechei* ATCC 49163 (as the case may be). Gene expression is twice as high as the reference strain is significant overexpression.

### Molecular typing method

#### Multi-locus sequence typing

Multi-locus sequence typing (MLST) of CR-ECC employing seven “housekeeping genes” (*dnaA, fusA, gyrB, leuS, pyrG, rplB*, and *rpoB*) was deduced by PCR and sanger sequenced following the protocol provided in the MLST website (https://pubmlst.org/organisms/enterobacter-cloacae) and re-confirmed by WGS. Each CR-ECC isolate was assigned to a specific sequence type (ST) upon uploading the aforesaid gene sequences and also the .fasta files generated by WGS on the MLST website (https://pubmlst.org/bigsdb?db=pubmlst_ecloacae_seqdef&l=1&page=sequenceQuery). The novel STs were confirmed by pubMLST curator. The phylogenetic relatedness and minimum spanning tree were elucidated using goeBURST and Phyloviz softwares.

#### Pulsed-field gel electrophoresis

The clonality among the CR-ECC isolates was deciphered by pulsed-field gel electrophoresis (PFGE) following standard protocol (39). Bacterial DNA was digested with *Xba*I restriction digestion enzyme (50U/plug) and PFGE was run for 21 hours at 6 V/cm potential with switch times of 5 and 35 s in CHEF-DRIII electrophoresis system (Bio-Rad Life Science Group, Hercules, CA, USA); using *E. cloacae* ATCC 13047 and *E. hormaechei* ATCC 700323 as positive controls and *XbaI*-digested *Salmonella* Braenderup H9812 as molecular weight ladder (40). FP-Quest Software, v4.5 (BioRad Laboratories, Hercules, CA, USA) was used to construct the dendrogram based on the Dice coefficient with a band position tolerance of 1.5%. The band patterns were analyzed as previously described (41).

#### Whole-genome shotgun sequencing and analysis

Genomic DNA of all the study strains (*n* = 29) was isolated by Qiagen DNeasy blood & tissue kit (Qiagen) and measured using Qubit 4.0 (Thermo Fisher Scientific, USA). The Nextera XT kit aided in DNA library preparation; and paired-end sequencing with a read length of 2 × 250 bp (base pairs) and a sequencing cycle of 2 × 501 was performed on the Illumina Novaseq 6000 platform following sequencing protocol v1.5 chemistry provided by the manufacturer (Illumina Inc, San Diego, CA). In addition, long-read sequences using SQK-LSK114 ligation Sequencing Kit were also generated through Nanopore MinION (Oxford, UK) to comprehend the genetic context of the *mcr*-9 and carbapenemase genes. Both Unicycler (github.com/rrwick/Unicycler) and CLC Workbench Premium 21.0 (Qiagen) software enabled *de novo* hybrid assembly using long and short raw reads. The quality of the assembled contigs was assessed by QUAST v5.0.2 (http://quast.sourceforge.net/). All erroneous mappings in the contigs produced during the assembly were rectified by PILON v1.23 (http://github.com/broadinstitute/pilon/releases/). The RAST server, accessible at (http://rast.nmpdr.org), facilitated the annotation of the genomes. The resulting .gbk file was visualized using Artemis, a genome viewer (Sanger UK). The SNAP Gene software v4.3.8 generated genomic images. The genome characteristics were determined by DFAST (https://dfast.ddbj.nig.ac.jp/). pubMLST (https://pubmlst.org/species-id) and ANI calculator enabled speciation of ECC (42). The online tool Resfinder 4.1 and PlasmidFinder 2.1 revealed the AMR genes and plasmid incompatibility (Inc) types. A phylogenetic tree based on wg-MLST was built cano-wgMLST software using (https://github.com/cenesis/cano-wgMLST).

## RESULTS

### Study isolates

Between January 2022 and December 2023, a total of 29 CR-ECC blood isolates comprising *E. hormaechei* (*n* = 23), *E. cloacae* (*n* = 5), and *E. kobei* (*n* = 1) were obtained from eight hospitals in Kolkata. Among the many subspecies of *E. hormaechei*, *E. hormaechei* subsp. *xiangfangensis* (*E*. *xiangfangensis; n* = 14) was the most frequently found CR-ECC in this study, followed by *E. steigerwaltii* (*n* = 5), *E. hoffmannii* (*n* = 3) and *E. oharae* (*n* = 1).

### Antimicrobial susceptibility test

Among the 29 CR-ECC strains, all were uniformly MDR to 19 of the 25 conventional antibiotics tested: imipenem (MIC_50_ 256 µg/mL), meropenem (MIC_50_ 512 µg/mL), ertapenem (MIC_50_ 256 µg/mL), doripenem (MIC_50_ 512 µg/mL), ceftaroline (MIC_50_ 128 µg/mL), cefepime (MIC_50_ 256 µg/mL), ceftazidime (MIC_50_ >1,024 µg/mL), cefotaxime (MIC_50_ >1,024 µg/mL), aztreonam (MIC_50_ 512 µg/mL), piperacillin-tazobactam (MIC_50_ 256/16 µg/mL), gentamicin (MIC_50_ 128 µg/mL), ciprofloxacin (MIC_50_ 256 µg/mL), levofloxacin (MIC_50_ 256 µg/mL), trimethoprim-sulfamethoxazole (MIC_50_ 128/304 µg/mL), chloramphenicol (MIC_50_ 512 µg/mL), tetracycline (MIC_50_ 512 µg/mL), ampicillin (MIC_50_ >1,024 µg/mL), ampicillin-sulbactam (MIC_50_ 512/64 µg/mL), and amoxycillin-clavulanic acid (MIC_50_ 256/32 µg/mL). High-level resistance (MIC_50_ >256/32 µg/mL) to ceftazidime-avibactam was found in 25 NDM-containing isolates. Colistin resistance (MIC range 32 to >256 µg/mL) was found in only six strains. The study isolates showed maximum susceptibility toward tigecycline (100%), followed by minocycline (85.7%), doxycycline (78.6%), and amikacin (75.7%).

### AMR genes

**Fig 1:**
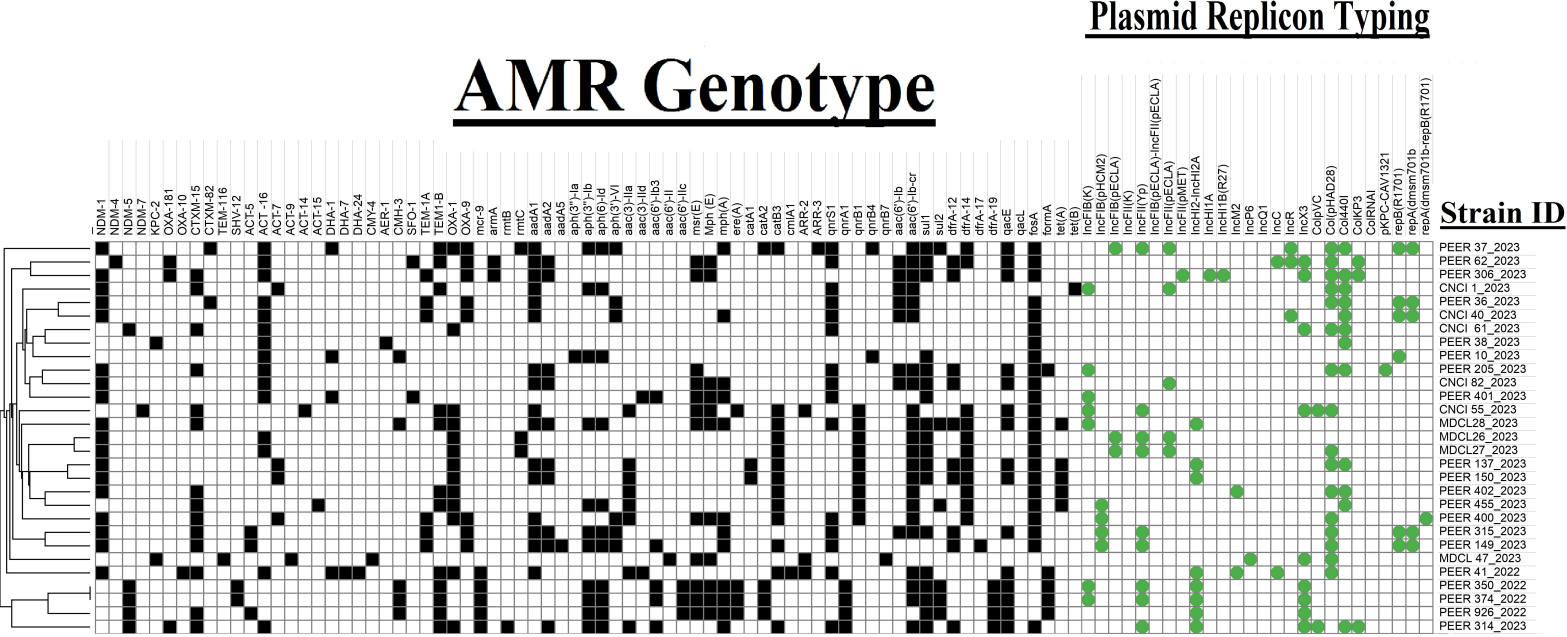
Heatmap of CR-ECC study isolates (*n* = 29) depicting respective AMR genes and plasmid replicon typing. Cluster analysis was performed on the heatmap using Euclidean distance. The filled boxes and circle represent the presence and no fill represents absence.

All CR-ECC isolates in this study showed MDR and produced a myriad of AMR genes conferring resistance to ≥9 different classes of antimicrobial agents. Out of the 29 strains, 86.20% (*n* = 25) of them tested positive for *bla*_NDM_ carbapenemase. Among the *bla*_NDM_ gene alleles, *bla*_NDM-1_ was predominant followed by *bla*_NDM-5_. In addition, another carbapenemase gene *bla*_OXA-181_ was found in two study isolates along with *bla*_NDM-4_ or *bla*_NDM-7_. Only two CR-ECC isolates (*E. xiangfangensis* and *E. kobei*) were *bla*_KPC-2_ producers. Two CR-ECC isolates did not show the presence of any carbapenemase genes in them. Four colistin-resistant (*n* = 4/6; 67%) isolates and one colistin-sensitive isolate were found to produce *mcr*-9 gene. Interestingly, *bla*_NDM-5_ was found to be significantly (*P* < 0.05) associated with isolates co-harboring *mcr*-9 genes. Besides the carbapenemases, all *mcr*-9-producing isolates also co-produced at least one variety of ampC (*bla*_ACT_ and/or *bla*_CMH-3_) and ESBL (*bla*_CTX-M-15_, *bla*_SHV-12_, or *bla*_SFO-1_) genes. Other AMR genes found in all the 29 study isolates are depicted in Fig. 1.

**Fig 2:**
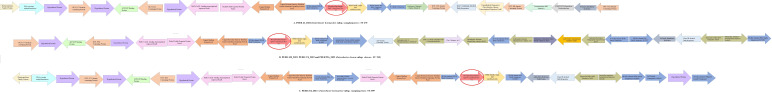
Genetic environment of *mcr-9* gene in CR-ECC study isolates.

WGS revealed identical *mcr*-9 genetic environments in four out of five study isolates which consisted of *rcnR-rcnA-pcoE-pcoS-IS903B-mcr-9-wbuC-IS26* (Fig. 2A and B); while one *E. xiangfangensis* isolate had slightly different genetic structure due to insertion of ISKpn24 (Fig. 2C). No difference was found between the genetic environment of colistin-resistant *mcr-*9 and colistin-susceptible *mcr*-9 study isolates.

Furthermore, several mutations as enlisted in Table S1 were found in pmrA, pmrB, and phoQ genes present in both *mcr*-9 producing and non-*mcr*-9-producing colistin-resistant isolates. The *mcr*-9-producing colistin-sensitive strain (PEER 41_2022) did not show any mutations in the aforesaid genes.

### Transferability of *mcr*-9 and carbapenemase gene

**Fig 3:**
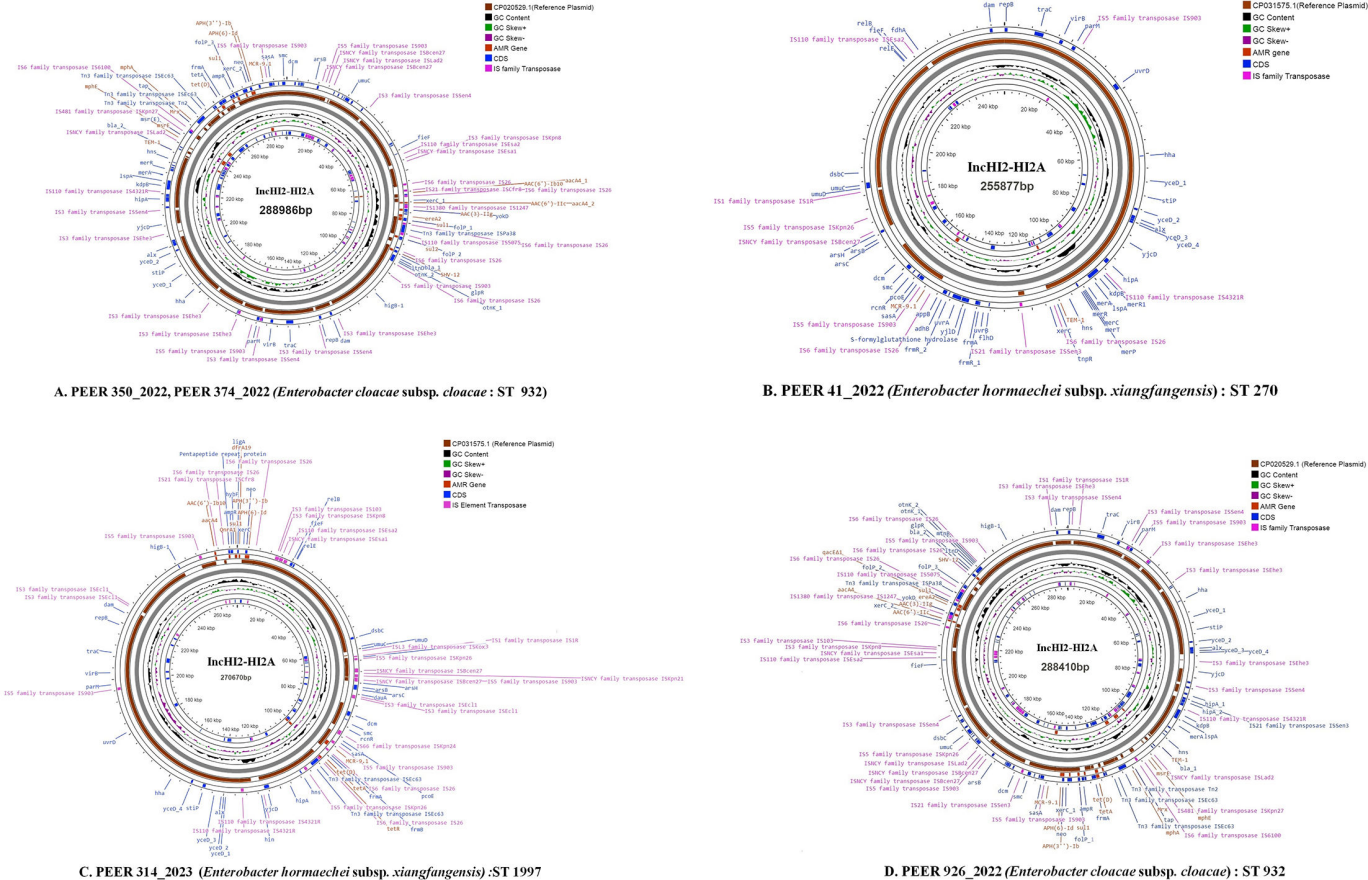
Circular map of the *mcr-9* carrying IncHI2-IncHI2A plasmids observed in the study isolates (A–D). *Enterobacter cloacae* (CP020529.1) and *Enterobacter hormaechei* (CP031575.1) are used as reference plasmids.

The *mcr*-9 gene present in five CR-ECC study isolates was found to be transferable via four different sized IncHI2-HI2A plasmids ranging from 255 to 289 kbp. Two isolates harbored identical plasmids. The genomic representation of these four plasmids based on difference in size, AMR genes and sequence types of the study strains has been presented in Fig. 3A through D. The transconjugants exhibited resistance to meropenem (MIC range 4–16 µg/mL), doripenem (MIC range 4–8 µg/mL) and ertapenem (MIC range 2–4 µg/mL) but showed intermediate susceptibility to colistin (MIC 2 µg/mL). Table S2 provides details of the MIC and frequency of transfer among transconjugants using different clinical strains of *Enterobacteriaecae* as recipients. The *mcr-9* gene was easily transferred to different genus of clinical strains highlighting its propensity of intra-genus dissemination and in turn increasing the burden of AMR. The study revealed simultaneous transmission of both *mcr*-9 and carbapenemase gene (*bla*_NDM-1_, *bla*_NDM-5_, and *bla*_OXA-181_) in the transconjugants irrespective of the selective agent used but via two different plasmids, i.e. IncHI2-HI2A and IncX3 (colistin-resistant *mcr-9* CR ECC study isolates) & IncHI2-IncHI2A and IncC (in case of colistin susceptible *mcr-9* CR-ECC study isolates) (Fig. 1).

### Colistin induction assay

**Fig 4:**
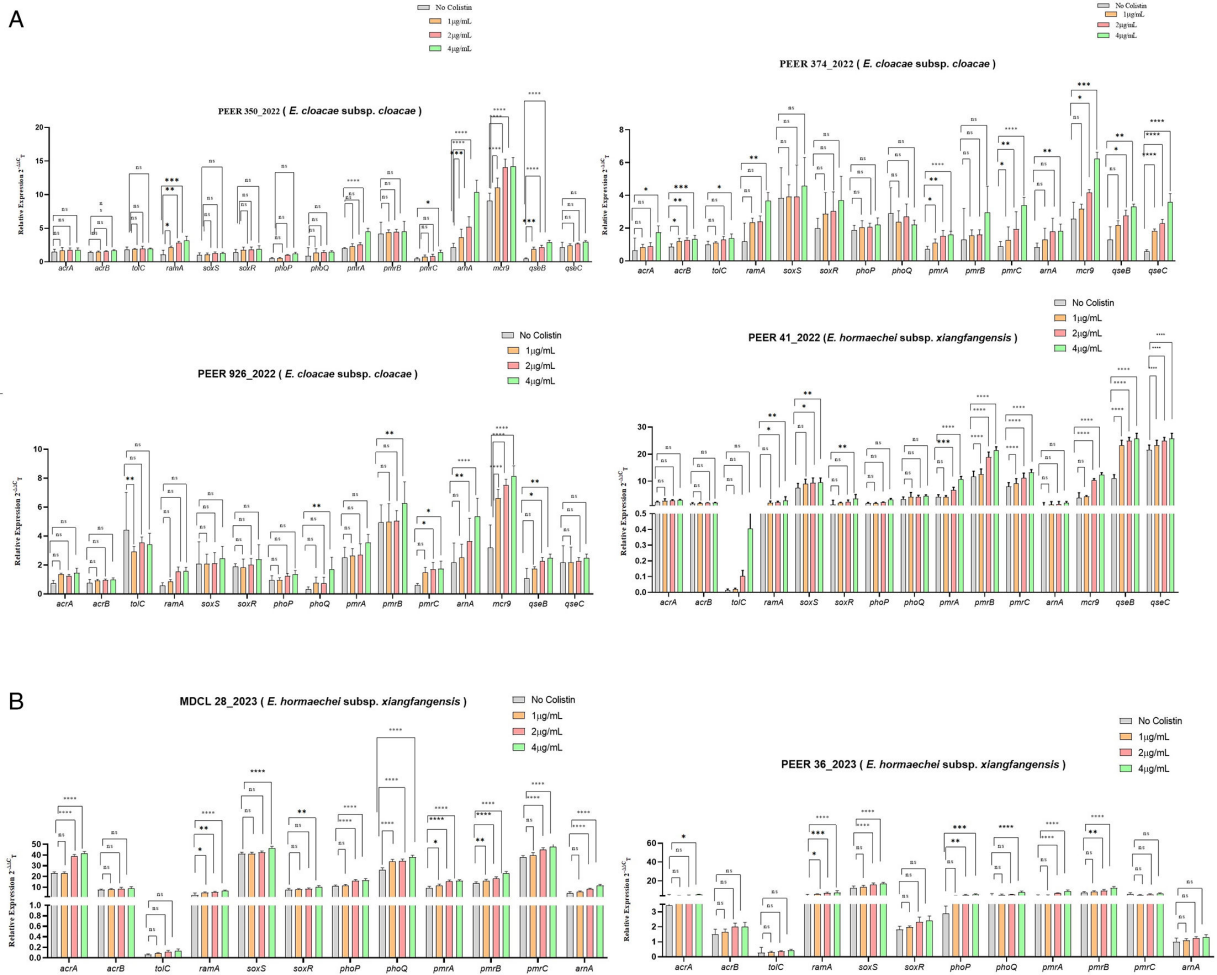
Analysis of gene expression in (A) *mcr-9* containing isolates and (B) non-*mcr-9* containing isolates, both with and without colistin exposure. Changes in mRNA levels have been measured using RT-qPCR. The data have been normalized to values for reference rpoB gene. Three independent triplicates were conducted, and the data presented are expressed as Mean ± SD. The asterisks symbolize varying levels of statistical significance as determined by ANOVA: *P ≤ 0.05; **P ≤ 0.005; ***P ≤ 0.001; ****P ≤ 0.0001; ns, not significant.

The inducibility of *mcr*-9 gene and its regulatory system *qseB* and *qseC* was validated by qPCR. Four *mcr*-9-producing (one colistin-sensitive and three colistin-resistant) wild type study isolates were exposed to increasing concentration of colistin (1, 2, and 4 µg/mL). In colistin-sensitive study isolate (PEER 41_2022), statistically significant increase in expression of *mcr*-9 and its regulatory *qseB* and *qseC* genes were seen following colistin induction (after induction of colistin 2 and 4 µg/mL concentration) in comparison to wild type. Parallelly, a ≥8-fold increase in MIC of colistin was observed following induction. On the other hand, although a significant increase in *mcr*-9 expression was seen following induction in the three colistin-resistant isolates (PEER 350_2022, PEER 374_2022, and PEER 926_2022), the change in MIC values was not significant (≤2-fold). A significant rise in the expression level of *qseB* was observed in all three *mcr-9*-producing CR-ECC isolates (PEER 350_2022, PEER 374_2022, and PEER 926_2022), while *qseC* was found to significantly increase in only PEER 374_2022 (Tables 1 and 2; Fig. 4) after colistin induction.

**TABLE 1:** Mechanism of colistin resistance in *mcr-9* and non-*mcr-9*-producing CR-ECC isolates: *mcr-9*-producing CR-ECC isolates^*a*^

Strain no/ ECC	Sequence types	MIC of colistin (µg/mL)	Colistin MIC presence of CCCP (µg/mL)	Colistin induction	MIC of colistin after colistin induction (µg/mL)	Colistin resistance mechanism														
						Efflux pump gene and their regulator^*c*^						Two component system Pathway and other gene^*d*^						*mcr-9* and its regulator^*e*^		
						acrA	acrB	tolC	ramA	soxS	soxR	phoP	phoQ	pmrA	pmrB	pmrC	arnA	mcr9	qseB	qseC
PEER 350/ *E*. *cloacae*	932	256	2	-	-	1.497 ± 0.356 ns	1.386 ± 0.183 **	1.785 ± 0.420 ns	1.05 ± 0.682 ****	1.032 ± 0.306 ns	1.384 ± 0.453 ns	0.503 ± 0.126 ****	1.304 ± 1.805 ns	2.009 ± 0.102 ****	4.134± 1.741 ns	0.443 ± 0.184 ****	2.118 ± 0.63 ****	9.076 ± 1.15 ***	0.434 ± 0.142 ****	2.176 ± 0.719 *
				1 µg/mL	256	1.701 ± 0.425 ns	1.505 ± 0.097 **	1.894 ± 0.098 ns	2.125 ± 0.218 ****	1.081± 0.218 ns	1.749 ± 0.425 ns	0.548 ± 0.123 ****	1.372± 0.585 ns	2.247 ± 0.448 ****	4.384± 0.377 ns	0.766 ± 0.255 ****	3.596 ± 1.26 ****	11.033 ± 1.417 ***	1.849 ± 0.326 ****	2.39 ± 0.298 *
				2 µg/mL	256	1.764 ± 0.305 ns	1.532 ± 0.123 **	1.932 ± 0.409 ns	2.816 ± 0.237 ****	1.243 ± 0.228 ns	1.820 ± 0.384 ns	0.98 ± 0.103 ****	1.39± 0.262 ns	2.578 ± 0.271 ****	4.452± 0.39 ns	0.792 ± 0.321 ****	5.192 ± 1.535 ****	14.036 ± 1.241 ***	2.15 ± 0.317 ****	2.652 ± 0.136 *
				4 µg/mL	512	1.775 ± 0.276 ns	1.692 ± 0.112 **	1.942 ± 0.090 ns	3.123 ± 0.685 ****	1.273 ± 0.147 ns	1.902 ± 0.467 ns	1.212 ± 0.209 ****	1.496± 0.153 ns	4.482 ± 0.501 ****	4.477± 1.525 ns	1.437 ± 0.301 ****	10.379 ± 1.774 ****	14.188± 1.340 ***	2.879 ± 0.328 ****	2.98± 0.181 *
PEER 374 */E*. *cloacae*	932	512	1	-	-	0.634 ± 0.747 **	0.843 ± 0.19 **	0.989 ± 0.209 **	1.191 ± 1.115 ****	3.83 ± 1.857 ns	1.994 ± 0.60 ns	1.862 ± 0.314 ns	2.902± 1.566 ns	0.703 ± 0.19 ****	1.286± 1.911 ns	0.817 ± 0.262 ****	0.889 ± 0.388 *	2.564 ± 1.014 ****	1.277 ± 0.80 ****	0.585 ±0.089 ****
				1 µg/mL	512	0.827± 0.173 **	1.173 ± 0.201 **	1.092 ± 0.069 **	2.336 ± 0.275 ****	3.908 ± 0.737 ns	2.862 ± 1.301 ns	2.029 ± 0.418 ns	2.377± 0.678 ns	1.083 ± 0.246 ****	1.545 ± 0.343 ns	1.286 ± 0.697 ****	1.286 ± 0.697 *	3.175± 0.287 ****	2.162 ± 0.282 ****	1.819± 0.126 ****
				2 µg/mL	512	0.863 ± 0.245 **	1.255 ± 0.149 **	1.302 ± 0.185 **	2.397 ± 0.347 ****	3.92 ± 1.94 ns	3.018 ± 1.197 ns	2.073 ± 0.222 ns	2.708± 0.762 ns	1.502 ± 0.358 ****	1.588± 0.309 ns	1.797 ± 0.804 ****	1.797 ± 0.804 *	4.165 ± 0.191 ****	2.757 ± 0.335 ****	2.273 ± 0.259****
				4 µg/mL	1,024	1.735 ± 0.398 **	1.330 ± 0.217 **	1.377 ± 0.263 **	3.658 ± 0.508 ****	4.578 ± 1.732 ns	3.704 ± 1.464 ns	2.194 ± 0.43 ns	2.207± 0.274 ns	1.584 ± 0.224 ****	2.949 ± 1.606 ns	1.802± 0.441 ****	1.802 ± 0.441 *	6.233 ± 0.393 ****	3.293 ± 0.171 ****	3.576 ± 0.53****
PEER 926/*E. cloacae*	932	64	0.25	-	-	0.724 ± 0.20 ****	0.741 ± 0.264 ns	3.772 ± 0.732 ns	0.577 ± 0.193 ****	2.061 ± 1.54 ns	1.879 ± 0.205 ns	0.948 ± 0.31 *	0.318 ± 0.168 **	2.502 ± 0.725 *	4.931± 1.219 ns	0.581 ± 0.92 ***	2.156 ± 1.355 **	3.185 ± 1.578 ****	1.055 ± 0.709 ****	2.152 ± 1.175 ns
				1 µg/mL	64	1.344 ± 0.082 ****	0.925 ± 0.084 ns	3.024 ± 0.259 ns	0.873 ± 0.115 ****	2.092 ± 0.656 ns	1.816 ± 0.592 ns	0.952 ± 0.17 *	0.771± 0.287 **	2.649 ± 0.487 *	5.048 ± 0.712 ns	1.486 ± 0.347 ***	2.527 ± 0.903 **	6.616 ± 0.604 ****	1.735 ± 0.140 ****	2.176 ± 1.057 ns
				2 µg/mL	64	1.238 ± 0.117 ****	0.951 ± 0.071 ns	3.633 ± 0.417 ns	1.559 ± 0.296 ****	2.109 ± 0.747 ns	2.006 ± 0.443 ns	1.227 ± 0.188 *	0.74± 0.315 **	2.694 ± 0.776 *	4.776± 1.015 ns	1.706 ± 0.466 ***	3.641 ± 1.585 **	7.521 ± 0.398 ****	2.252 ± 0.277****	2.252 ± 0.277ns
				4 µg/mL	128	1.452 ± 0.326 ****	0.99 ± 0.116 ns	3.692 ± 0.531 ns	2.462 ± 0.827 ****	1.579 ± 0.286 ns	2.399 ± 1.001 ns	1.355 ± 0.26 *	1.714± 1.30 **	3.543 ± 0.563 *	6.275± 1.474 ns	1.732 ± 0.534 ***	5.344 ± 1.263 **	8.125 ± 0.730 ****	2.489 ± 0.277 ****	2.489 ± 0.262ns
*E. cloacae* ATCC 13047	1	0.125	ND	ND	ND	1	1	1	1	1	1	1	1	1	1	1	1	1	1	1
*PEER 41_2023/E. hormaechei* subsp. *xiangfangensis*	270	0.5	0.125	-	-	2.324 ± 0.277 ns	1.69 ± 0.378 *	0.013 ± 0.008 ****	0.525 ± 0.415 **	7.448 ± 1.759 ns	1.391 ± 1.571 *	1.862 ± 0.317 ****	3.315 ± 0.862 ns	4.143 ± 0.861 ****	11.713 ± 1.967 ****	7.385 ± 1.865 ****	1.316 ± 1.743 ns	3.812 ± 1.903 ****	11.118 ± 1.297 ****	21.621 ± 1.709 **
				1 µg/mL	8	2.713± 0.797 ns	1.918 ± 0.183 *	0.019 ± 0.006 ****	2.06 ± 0.385 **	8.98 ± 1.768 ns	1.961 ± 0.268 *	1.891 ± 0.201 ****	4.18± 1.633 ns	4.168 ± 0.491 ****	12.518 ± 1.981 ****	9.16 ± 1.768 ****	0.903 ± 1.198 ns	4.357 ± 0.348****	23.203 ± 1.942****	23.203 ± 1.942**
				2 µg/mL	64	2.836± 0.33 ns	1.943 ± 0.106*	0.103 ± 0.036 ****	2.202 ± 0.113 **	9.429 ± 1.77 ns	2.417 ± 0.65 *	2.153 ± 0.167 ****	4.262± 0.538 ns	6.712 ± 1.052 ****	18.929± 1.818 ****	11.153 ± 1.823 ****	1.441 ± 1.036 ns	10.338 ± 0.595****	24.936 ± 1.252****	24.936 ± 1.252**
				4 µg/mL	64	3.095± 0.161 ns	2.072 ± 0.078*	0.405 ± 0.251 ****	2.774 ± 1.498 **	9.577 ± 1.596 ns	3.596 ± 1.491 *	3.164 ± 0.458 ****	4.483± 0.485 ns	10.629 ± 1.151 ****	21.457 ± 1.255 ****	13.157 ± 1.12 ****	2.035 ± 0.493 ns	12.330± 0.810****	25.703 ± 1.958****	27.096 ± 1.942**
*E. hormaechei* ATCC 49163	1	0.125	ND^*b*^	ND	ND	1	1	1	1	1	1	1	1	1	1	1	1	1	1	1

^*a*^ Three sets of independent experiments were performed in triplicate. The values of qPCR fold change have been represented as mean ± SD. The asterisks indicate statistical significance at different levels by ANOVA: *P ≤ 0.05; **P ≤ 0.005; ***P ≤ 0.001; ****P ≤ 0.0001; ns, not significant. Every significant difference was observed in comparison to individual wild-type strains to which colistin antibiotic was not added.

^*b*^ ND, not determined.

^*c*^ *acrA, acrB, tolC, ramA, soxS, soxR.*

^*d*^ *phoP*, *phoQ*, *pmrA*, *pmrB*, *pmrC*, *arnA.*

^*e*^ *mcr-9*, *qseB*, *qseC*.

**TABLE 2:** Mechanism of colistin resistance in *mcr-9* and non-*mcr-9-*producing CR-ECC isolates: Non *mcr-9*-producing CR-ECC isolates^*a*^

Strain No/ECC	Sequence types	MIC of colistin (µg/mL)	Colistin MIC presence of CCCP (µg/mL)	Colistin induction	MIC of colistin after colistin induction (µg/mL)	Colistin resistance mechanism											
						Fold changes of efflux pump and their regulator^*c*^						Fold changes of two-component system pathway and other gene^*d*^					
						acrA	acrB	tolC	ramA	soxS	soxR	phoP	phoQ	pmrA	pmrB	pmrC	arnA
PEER36_2023 / *E. hormaechei* subsp. *xiangfangensis*	66	8	0.5	-	-	3.42 ± 1.817 ns	1.510 ± 0.326 **	0.282 ± 0.352 ns	4.32± 1.262 **	12.566 ± 1.891 ****	1.826 ± 0.208 **	2.888 ± 0.495 ****	4.361± 1.923 ***	3.91 ± 1.007 ****	7.505± 1.068 ****	4.637 ± 0.410 ns	0.998± 0.245 *
				1 µg/mL	32	3.607 ± 0.77 ns	1.663 ± 0.181 **	0.29 ± 0.074 ns	5.896 ± 0.32 **	13.182 ± 1.947 ****	1.964 ± 0.093 **	3.814 ± 0.441 ****	4.874 ± 0.856 ***	4.079± 0.858 ****	8.43 ± 1.092 ****	4.557 ± 0.483 ns	1.096± 0.108 *
				2 µg/mL	128	4.041 ± 0.838 ns	1.991 ± 0.25 **	0.36 ± 0.047 ns	6.732 ± 1.172**	15.886 ± 1.78 ****	2.322 ± 0.320 **	4.985 ± 0.489 ****	5.06 ± 0.542***	6.541± 0.68 ****	9.47 ± 1.007 ****	5.798 ± 0.641 ns	1.231± 0.112 *
				4 µg/mL	512	5.081 ± 0.562 ns	2.005 ± 0.29 **	0.41 ± 0.085 ns	7.526 ± 1.894 **	16.633 ± 1.69 ****	2.423 ± 0.295 **	5.028 ± 1.069 ****	7.755 ± 1.927 ***	9.025± 1.169 ****	12.41 ± 1.31 ****	6.787 ± 0.562 ns	1.319± 0.155 *
MDCL 28_2023/ *E. hormaechei* subsp. *xiangfangensis*	Novel	64	2	-	-	22.834 ± 1.54 ****	7.620 ± 0.580 ns	0.059± 0.014 ***	2.715± 3.963 ***	40.996 ± 1.609 ****	7.554 ± 1.234 **	10.835 ± 1.084 ****	26.098 ± 1.054 ****	9.465± 1.315 ****	13.557 ± 1.437 ****	37.891 ± 1.519 ****	4.238± 1.696 ****
				1 mg/mL	128	23.089 ± 1.19 ****	7.729 ± 0.884 ns	0.083 ± 0.016 ***	4.782 ± 0.878 ***	41.157 ± 1.463 ****	8.092 ± 0.218 **	11.296 ± 1.28 ****	34.025 ± 1.872 ****	11.613± 1.266 ****	16.061 ± 1.17 ****	41.009 ± 1.758 ****	5.602± 0.868 ****
				2 mg/mL	512	38.753 ± 1.749 ****	8.476 ± 1.411 ns	0.118 ± 0.235 ***	5.411 ± 0.703 ***	42.408 ± 1.374 ****	8.521 ± 0.856 **	15.773 ± 1.357 ****	34.532 ± 1.744 ****	15.579± 1.129 ****	18.024 ± 1.655 ****	44.341 ± 0.993 ****	8.158± 0.951 ****
				4 µg/mL	>1,024	41.447 ± 1.896 ****	8.766 ± 1.738 ns	0.132 ± 0.035 ***	6.470 ± 0.761 ***	46.533 ± 1.578 ****	9.995 ± 1.54 **	16.452 ± 1.57 ****	37.598 ± 1.612 ****	15.83± 1.103 ****	23.074 ± 1.626 ****	46.760 ± 0.972****	11.491± 1.244 ****
*E. hormaechei* ATCC 49163	1	0.125	ND^*b*^	ND	ND	1	1	1	1	1	1	1	1	1	1	1	1

^*a*^ Three sets of independent experiments were performed in triplicate. The values of qPCR fold change have been represented as Mean ± SD. The asterisks indicate statistical significance at different levels by ANOVA: *P  ≤  0.05; **P  ≤  0.005; ***P  ≤  0.001; ****P  ≤  0.0001; ns, not significant. Every significant difference was observed in comparison to individual wild-type strains to which colistin antibiotic was not added.

^*b*^ ND, not determined

^*c*^ *acrA*, *acrB*, *tolC*, *ramA*, *soxS*, *soxR*

^*d*^ *phoP*, *phoQ*, *pmrA*, *pmrB*, *pmrC*, *arnA*

In *mcr*-9-producing colistin-resistant study isolates, no significant changes were observed in the expression level of efflux pump genes *acrAB-tolC* and its regulator *ramA* and *soxS* by RT-PCR. However, in PEER 926_2022, the addition of 11 amino acids (MINQEAEGESS) was observed in ramA along with 36 amino acid deletion (MNKNRGLTPLAVVLMLSGSLTLTGCDDK QAQQGAQQ) at positions 1–36 of acrA. In the other two isolates namely, PEER 350_2022 and PEER 374_2022, no mutations were observed except the 36 amino acid deletion in acrA as previously described. The changes in the expression level of efflux pump genes remained insignificant even after colistin induction. In *mcr*-9- producing colistin-sensitive isolate high level of expression of *acrA* gene alone was seen in both wild (2.34-fold) and induced strains (3.09-fold) along with the same 36 amino acid deletion. The *acrA, soxS,* and *ramA* genes were found to be overexpressed in two non-*mcr*-9-producing colistin-resistant CR-ECC isolates when compared to colistin-susceptible ATCC control strains (Tables 1 and 2). The concurrent rise in expression level and MIC of colistin was observed following colistin exposure (Tables 1 and 2). The change in MIC on day 1 after exposure was found to be stable even after 15 days of exposure (MIC was checked on day 1, day 7, and day 15 following induction as given earlier). While several mutations {acrA (Q234T), acrB (H1047P, D663A, A699E), tolC (N79A, E105A, L261Q, M298Q, Q432L, M433A, D457V, deletion of amino acid SPAS at positions 481–484), and ramA (E83A and 11 amino acid MINQEAEGESS deletion at positions 1–11)} were found in one isolate, only a 36 amino acid deletion in acrA was found in the other isolate.

### Role of a two-component system in colistin resistance

A significantly high expression of all four genes *phoP, phoQ, pmrA,* and *pmrB* of the two-component system was observed in both wild and induced non-*mcr*-9-producing colistin-resistant (Tables 1 and 2). Another important gene *arnA, a* part of the arn operon which is responsible for lipid A modification of the cell and in turn contributing to colistin resistance was found to be upregulated in one non-*mcr-9*-producing colistin-resistant isolate (MDCL28_2023) (Tables 1 and 2). But in the case of colistin-sensitive *mcr-9*-producing isolate (PEER41_2022) *pmrA-pmrB* system was found to be highly expressed (Tables 1 and 2). Another important gene *arnA, a* part of the arn operon which is responsible for lipid A modification of the cell and in turn contributing to colistin resistance was found to be upregulated in one non-*mcr-9*-producing colistin-resistant isolates (Tables 1 and 2). But in the case of colistin-sensitive *mcr-9*-producing isolate (PEER41_2022) *pmrA-pmrB* system was found to be highly expressed (Tables 1 and 2).

### PFGE

**Fig 5:**
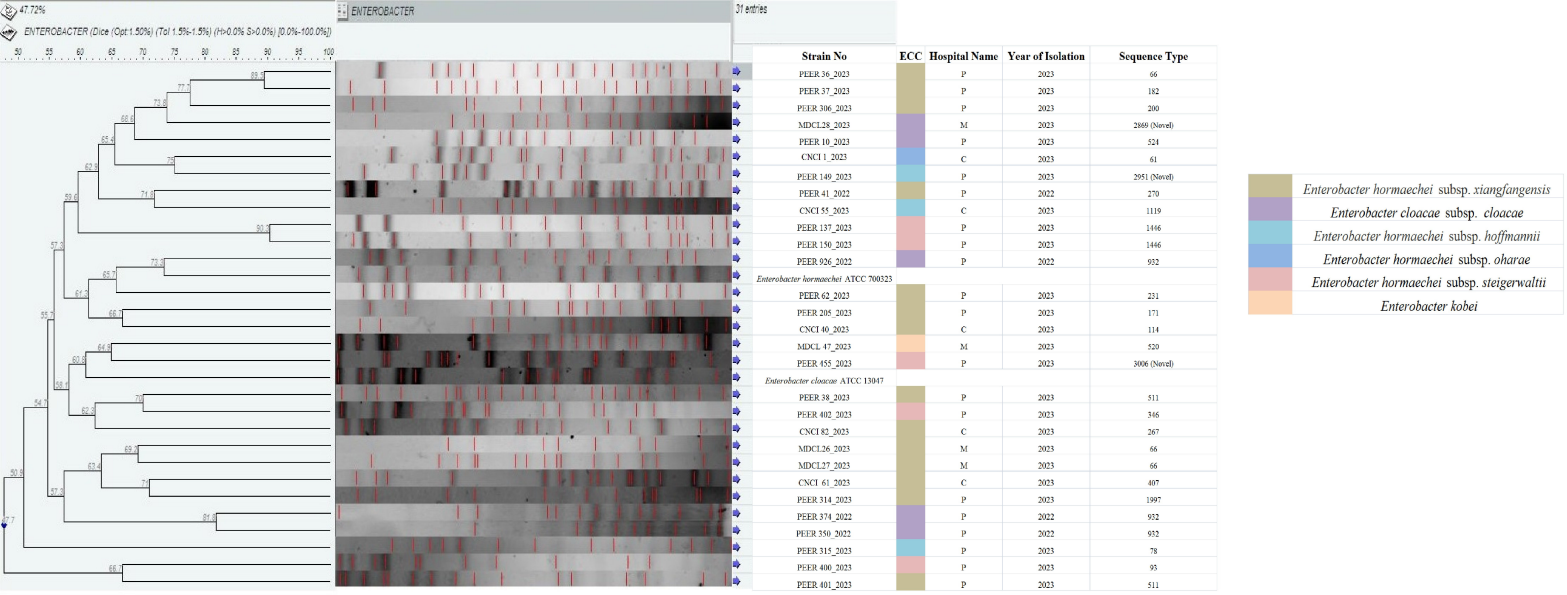
Dendrogram of PFGE cluster analysis of *Xba*I-digested patterns of (*n* = 29) carbapenem non-susceptible *Enterobacter cloacae* complex (ECC) isolates.

PFGE analysis of 29 CR-ECC isolates revealed heterogeneity (similarity coefficient of 47.72%) among them. Neither any correlation existed among pulsotypes, STs or AMR-profiles nor any difference between *mcr-9-*producing and non-*mcr-9-*producing colistin-resistant CR-ECC strains was observed (Fig. 5). Any association between the hospital and strain pulsotypes was also not found. A broad spectrum of infection reservoirs and a higher risk of dissemination are both suggested by the phylogenetic diversity of circulating CR-ECC strains.

### MLST

**Fig 6:**
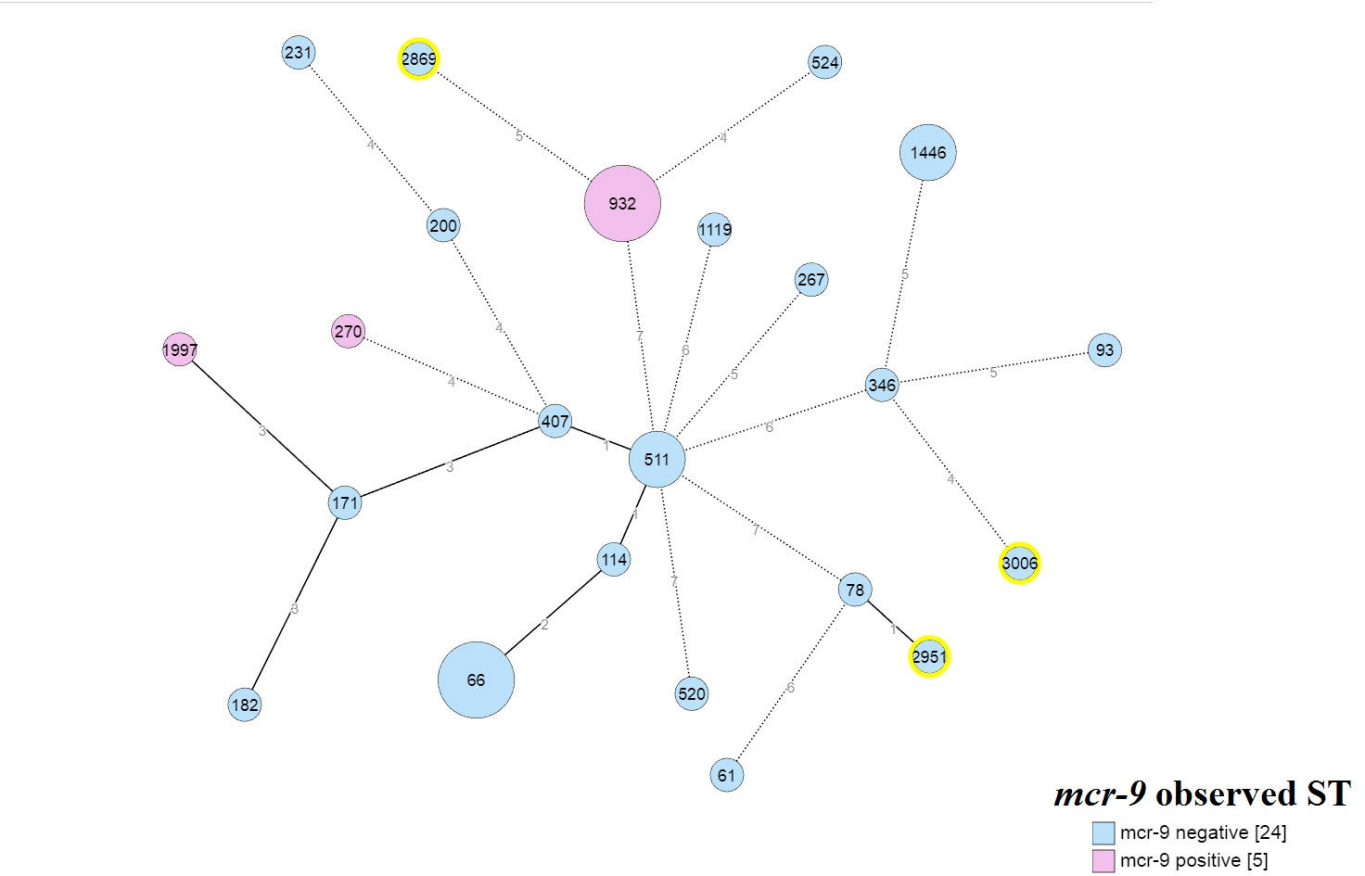
Minimum spanning tree (MST) based on MLST allelic profile: Pictorial depiction of genetic relationship of different sequence types (ST) of ECC-CR clinical isolates found in this study by goeBURST and PHYLOViZ 2.0. Each kurtosis or node (circle) corresponds to one ST designated by a particular number, and the size of the circle is proportional to the number of isolates (genomes). The blue color kurtosis represents the STs found in mcr-9-positive study isolates while the pink color indicates The STs found in mcr-9-negative isolates. The numbers on the Branches represent the allelic differences. Branches with a length greater than three locus variants are outlined in dashes. All the STs obtained in this study (India) are validated and curated by the pubMLST curator, and this is now available at the pubMLST website. The kurtosis circles with yellow halos (ST2869, ST2951, and ST3006) depict the novel STs discovered in this study.

MLST segregated the 29 CR-ECC study isolates into 23 distinct STs: ST66, ST932 (*n* = 3 each); ST511, ST1446 (*n* = 2); ST61, ST78, ST93, ST114, ST171, ST182, ST200, ST231, ST270, ST 346, ST407, ST520, ST524, ST1119, ST1997, ST2869, ST2951, and ST3006 (*n* = 1 each) (Fig. 4). All *mcr-9*-producing *E. cloacae* subsp. *cloacae* isolates (*n* = 3) belonged to ST932; while the *mcr-9*-producing *E. hormaechei* subsp. *xiangfangensis* belonged to ST1997 (colistin-resistant) and ST270 (colistin-susceptible). The colistin-resistant but non-*mcr-9-*producing strains were assigned to ST66 (*E. xiangfangensis*) and a novel ST2869 (*E. cloacae*). Analysis of the molecular epidemiological relationships between different STs by goeBURST revealed that all the STs were singletons except ST511 which varied from both ST407 and ST114 at a single but different locus. Interestingly, ST114 and the novel ST2951 seemed to have evolved from ST66 (by double locus variation) and ST78 (by single locus variation), respectively (Fig. 6).

### wg-MLST

**Fig 7:**
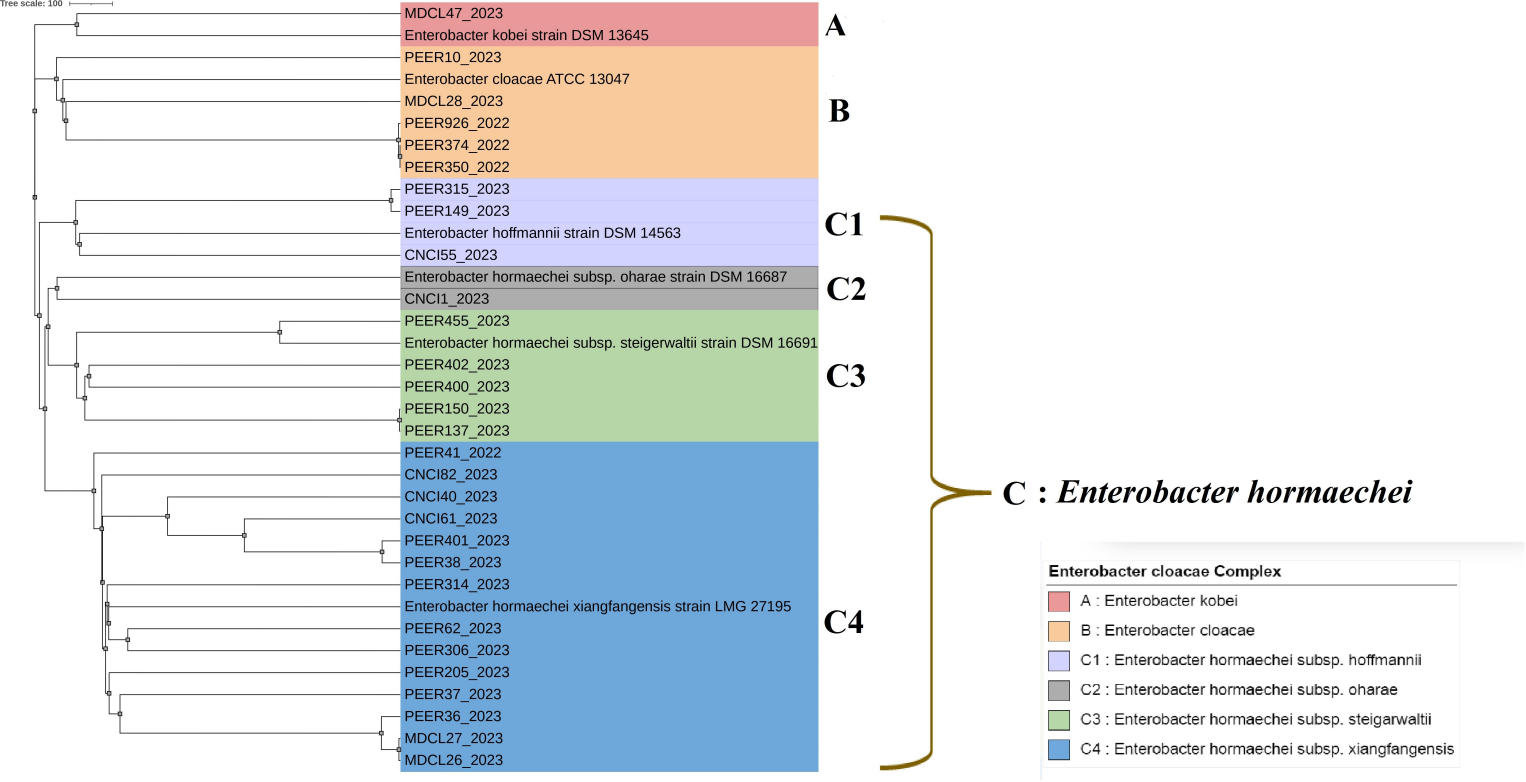
Whole-genome multi-locus sequence typing (wg-MLST)-based phylogenetic tree of CR-ECC strains. The reference genomes of respective ECC type (*E. hormaechei* subsp. *xiangfangensis* LMG 27195 accession no. CP017183; *E. hormaechei* subsp. *steigerwaltii* DSM 16691 accession no. CP017179; *E. hormaechei* subsp. *hoffmanii* DSM 14563 WJWQ00000000; *E. hormaechei* subsp. oharae DSM 16687 accession no CP017180; *E. cloacae* ATCC 13047 accession no. CP001918; and *E. kobei* DSM 13645 accession no. CP017181 were used to perform the wg-MLST.

Three distinct clusters A, B, and C representing *E. kobei*, *E. cloacae, and E. hormaechei,* respectively, were generated by wg-MLST cladogram (Fig. 7). *E*. *hormaechei* subsp. *hoffmannii*, subsp. *oharae*, subsp. *steigerwaltii*, subsp. *xiangfangensis* and formed separate sub-clusters C1, C2, C3, and C4 within cluster C.

## DISCUSSION

This study gives a comprehensive description of the mechanism of resistance and molecular subtypes of colistin-resistant CR-ECC blood isolates emerging in India. Earlier, the colistin-resistant *E. cloacae* was reported from Southern India, but neither genome characteristics nor mechanism of colistin resistance was investigated (43). To the best of the authors’ knowledge, this is the first study to report the emergence of the *mcr*-9 gene in CR-ECC isolates from India.

All the *mcr*-9-producing study isolates were multidrug resistant with or without colistin resistance. Unlike previous studies (19), the *mcr*-9-producing colistin-resistant study isolates showed high MIC values for colistin (≥64 µg/mL). All the *mcr*-9-producing colistin-resistant study isolates exhibited resistance to polymyxin B (MIC 32–128µg/mL), whereas the *mcr*-9- producing colistin-sensitive isolate was also susceptible to polymyxin B indicating that resistance to colistin and polymyxin B in our study isolates were inter-linked. The presence of *mcr*-9-producing colistin-sensitive strains in circulation often leads to an underestimation of the global prevalence rate of these genes as they frequently evade detection. Furthermore, colistin resistance can be induced in them by exposure to a subminimal amount of colistin in the environment as indicated by the colistin induction assay in this study (Tables 1 and 2). The spread of such strains is daunting with respect to public health.

The role of the regulatory genes *qseB-qseC* in colistin induction is debatable. The presence of *qseC* and *qseB* genes downstream of *mcr*-9 is thought to be involved in the induction of *mcr*-9 expression at sub-inhibitory concentrations of colistin, according to some researchers (18, 19). However, other studies have demonstrated that the change in MIC of colistin after induction is unrelated to these genes (19). In our study, we found that the expression levels of *mcr*-9, *qseB,* and *qseC* were significantly elevated in *mcr*-9-producing colistin-sensitive and *mcr*-9-producing two colistin-resistant isolates after colistin induction (Tables 1 and 2); however, WGS analysis of the genetic environment of *mcr*-9 gene resident on the IncHI2-HI2A plasmid showed the absence of *qseB-qseC* genes downstream of *mcr*-9. The *mcr*-9 genetic environment (IS903B-mcr-9-wbuC-IS26) (Fig. 2) was found to be consistent with those reported in ECC from China and the USA (7, 16).

Colistin resistance is often associated with mutations in genes that are involved in the pmrAB or phoPQ two-component regulatory system as reported in earlier studies (44). Consistent with earlier reports (6, 45), the colistin-resistant study isolates also showed several mutations (Table S1), but these mutations were random and were not previously reported except I294T in pmrB colistin-resistant *mcr-9* containing *E. cloacae* subsp. *cloacae*(*n* = 3) study isolates and T271Q in pmrB in one colistin-resistant non *mcr-9 E. hormaechei* subsp. *xiangfangensis* study isolate (46). Unlike previous reports, species-specific mutations were not found in the study isolates (47). Validation of the role of these observed amino acid changes in *phoPQ* and *pmrAB* of study isolates in colistin resistance is beyond the scope of this study.

The role of efflux pump (*acrAB-tolC*) and its regulatory genes (*ramA* and *soxS*) in colistin resistance was investigated in the study isolates and overexpression of *acrA*, *ramA,* and *soxS* genes in non-*mcr*-9-producing colistin-resistant isolates was found (Tables 1 and 2) indicating an active role of the efflux pump in mediating colistin resistance in these isolates, consistent with earlier research (13). The presence of an energy-dependent efflux of colistin by the efflux pump gene, *acrB,* and *ramA* in *K*. *pneumoniae* was also previously reported (48, 49). None of the non-*mcr-*9-producing isolates exhibited *acrB* overexpression, a finding that aligns with previous research (11).

Analysis of the gene sequences of the efflux pump genes revealed a number of mutations in the amino acid sequences of *acrA, acrB, tolC,* and *ramA* of the study isolates (as previously described) but it is difficult to ascertain their role in gene expression. This is because although we found an upregulation of *soxS* gene in the non-*mcr*-9-producing colistin-resistant study isolates, the sequences of *soxS* and its regulator, *soxR*, were unaltered among all isolates. Similarly, a 36 amino acid deletion (MNKNRGLTPLAVVLMLSGSLTLTGCDDKQAQQGAQQ) at positions 1–36 in acrA was found in all isolates irrespective of their over/under expression. The two-component system *phoP/Q* and *pmrA/B* was also found to be more active in non-*mcr*-9-producing colistin-resistant isolates which is similar to previous research. The *arnA* gene (arn operon) was also found to be more active in one non-*mcr*-9-producing colistin-resistant isolate (Tables 1 and 2). The *phoP/Q* are positive regulators of the arn operon (8, 9), so upregulation of *phoP/Q* genes leads to upregulation of the arn operon which, in turn, leads to colistin resistance. An interplay of the efflux pump and a two-component system might play a major role in non-*mcr*-9-producing colistin-resistant isolates in our study. No homologs of the recently described transmembrane protein, Enterobacter colistin resistance (*ecr*) gene, which causes colistin resistance by activating the *arnBCADTEF* operon via the PhoPQ system (12) was found in the study isolates.

A large variety of sequence types (Fig. 6) were identified in this study, most of which have already been reported from China and the USA (47, 50, 51). However, ST2869 and ST2951 are the novel ST being reported for the first time in this study. No specific STs exhibited dominance in this study, although ST predominance is known to vary with countries/regions (50, 51). A particular ST is typically associated with an antimicrobial resistance (AMR) phenotype. However, this study did not observe any such correlation. The colistin-resistant/*mcr*-9-producing isolates typed into ST1997, ST932, ST66, ST78, and ST270 were not previously reported from India. Out of the above five, ST66 and ST78 have been globally linked to ESBLs in ECC and are known to harbor the *bla*_CTX-M-15_ gene (50). Surprisingly, two of the three *E. xiangfangensis* study isolates typed into ST66 did not harbor *bla*_CTX-M-15_ gene but all three produced bla_NDM-1_ carbapenemase. The association of ST66/ST78 with colistin resistance and *bla*_NDM-1_ as found in this study is worrisome and was not previously documented. Unlike previous reports from Ukraine (52), the three *E. cloacae* ST932 study isolates were found to co-harbor *bla*_NDM-5_ and *mcr-9* (colistin resistant) genes in addition to ESBLs *bla*_CMH-3_ and *bla*_CTX-M-15/SHV-12_. The emergence and rapid dissemination of this ST932 poses a great threat to healthcare as it renders simultaneous resistance to three major antimicrobial classes namely colistin, carbapenem, and cephalosporins. Among the different molecular typing methods used in this study, MLST was far less discriminatory than PFGE as isolates with the same STs exhibited different pulsotypes in PFGE indicating the presence of multiple repositories of CR-ECC in this particular region and hence multiple sources of infection in humans (Fig. 5). However, in comparison to PFGE, wg-MLST was more effective in clustering CR-ECC study isolates of same subspecies into same clades (Fig. 7).

This study is not without limitations. It does not depict the true magnitude of the emerging colistin resistance or *mcr*-9 in ECC pan-India because of the short study period and restriction to the Kolkata region. Furthermore, not all mechanisms of colistin resistance could be investigated in the study isolates. Assessing the role of several mutations found in efflux pump and two-component system genes in the study isolates was beyond the scope of this study.

### Conclusion

Our study provides physicians with a cautionary message regarding the potential occurrence of a public health crisis resulting from the simultaneous transmission of *mcr-9* and carbapenemase (*bla*_NDM-1_, *bla*_NDM-5_, and *bla*_OXA-181_) genes through conjugative plasmids within healthcare establishments. Thorough surveillance of IncHI2-HI2A plasmids is essential as it is the main catalyst for the spread of *mcr-9* in CR-ECC. It is recommended to conduct regular screening of colistin-sensitive CR-ECC isolates due to their role as silent carriers of the *mcr-9* gene, which is found to be upregulated in response to colistin-induced stress in the surrounding environment.

## Data Availability

All genome sequences have been submitted to the NCBI database under BioProject numbers PRJNA1017358 and PRJNA957587.

## SUPPLEMENTAL MATERIAL

Table S1 spectrum.01542-24-s0001.docx Mutations observed in the mcr-9 CR-ECC strains and non -mcr-9 colistin resistant CR-ECC.

Table S2 spectrum.01542-24-s0002.doc Minimum Inhibitory Concentration (MIC) of donor, recipient, transconjugants and frequency of transfer among transconjugants.

Table S3 spectrum.01542-24-s0003.doc Assembly metrics, biosample, and accession numbers of the CR-ECC study isolates.

ASM does not own the copyrights to Supplemental Material that may be linked to, or accessed through, an article. The authors have granted ASM a non-exclusive, world-wide license to publish the Supplemental Material files. Please contact the corresponding author directly for reuse.
