# Supplementary material for: Emergence of concurrently transmissible mcr-9 and carbapenemase genes in bloodborne colistin-resistant Enterobacter cloacae complex isolated from ICU patients in Kolkata, India
Source: Microbiol Spectr. 2025 Feb 6;13(3):e01542-24. doi: 10.1128/spectrum.01542-24 (PMC11878022; doi:10.1128/spectrum.01542-24)
Supplement: Table S1 — Mutations observed in the mcr-9 CR-ECC strains and non -mcr-9 colistin resistant CR-ECC. [file spectrum.01542-24-s0001.docx]

**Table S1. Mutations observed in the *mcr-9* CR-ECC strains and non *-mcr-9* colistin resistant CR-ECC by WGS**

| Strain with characteristics | phoP | phoQ | PmrA | pmrB |
| --- | --- | --- | --- | --- |
| PEER 350_2022 Col^R^+*mcr-9* (*Enterobacter cloacae* subsp. *cloacae*) | Wild Type | MKRL (1-4 position amino acid) deletion | Wild Type | **I295T**(1) |
| PEER 374_2022  Col^R^ +*mcr-9* (*Enterobacter cloacae* subsp. *cloacae*) | Wild Type | MKRL (1-4 position amino acid) deletion | Wild Type | **I295T**(1) |
| PEER 926_2022  Col^R^+*mcr-9*(*Enterobacter cloacae* subsp. *cloacae*) | Wild Type | MKRL (1-4 position amino acid) deletion | Wild Type | **I295T**(1) |
| MDCL 28_2023  Col^R^  (*Enterobacter hormaechei* subsp. *xiangfangensis*) | Wild Type | MKRL (1-4 position amino acid) deletion, K106V, R108S, I141K, D151A, L168P, M190R, V415Q, L483T | R143C, Q145L, S218R | E265T, T271Q, T280I, G347E |
| PEER 36_2023  Col^R^ (*Enterobacter hormaechei* subsp*.* *xiangfangensis*) | Wild Type | L483V | Wild Type | Wild Type |
| PEER 41_2022  Col^S^+*mcr-9*(*Enterobacter hormaechei* subsp. *xiangfangensis*) | Wild Type | Wild Type | Wild Type | Wild Type |

Mutations in genes conferring Colistin resistance in *E. cloacae* complex. study isolates (pmrA, pmrB, phoP, and phoQ) were identified by aligning their nucleotide and amino acid sequences with reference genome sequences of *E. cloacae* ATCC13047 (GenBank accession number: [CP001918](https://www.ncbi.nlm.nih.gov/datasets/genome/GCF_000025565.1/)) and *E. hormaechei* subsp*. xiangfangensis* LMG 27195 (GenBank accession number: [CP017183](https://www.ncbi.nlm.nih.gov/nuccore/CP017183)) in the NCBI database.

**Bold** – Reported already (1)

1. Puljko A, Barišić I, Dekić Rozman S, Križanović S, Babić I, Jelić M, Maravić A, Udiković-Kolić N. 2024. Molecular epidemiology and mechanisms of carbapenem and colistin resistance in Klebsiella and other Enterobacterales from treated wastewater in Croatia. Environment International 185:108554.
