# Supplementary material for: Emergence of concurrently transmissible mcr-9 and carbapenemase genes in bloodborne colistin-resistant Enterobacter cloacae complex isolated from ICU patients in Kolkata, India
Source: Microbiol Spectr. 2025 Feb 6;13(3):e01542-24. doi: 10.1128/spectrum.01542-24 (PMC11878022; doi:10.1128/spectrum.01542-24)
Supplement: Table S2 — Minimum Inhibitory Concentration (MIC) of donor, recipient, transconjugants and frequency of transfer among transconjugants. [file spectrum.01542-24-s0002.doc]

**Table S2. Minimum Inhibitory Concentration (MIC) of donor, recipient, Transconjugants and frequency of transfer among transconjugants**

| Donor | Recipient | Trans  Conjugant (TC) | Frequency of Transfer* | Minimum Inhibitory Concentration(MIC) (µg/mL) | | | | | | | | | |
| --- | --- | --- | --- | --- | --- | --- | --- | --- | --- | --- | --- | --- | --- |
|  |  |  |  | MEM | DOR | ERT | COL | CFP | CAZ | AZT | CAT | TET | GMN |
| PEER 41_2022 | - | - | - | 128 | 64 | 64 | 128 | 128 | 256 | 128 | 512 | 128 | 128 |
| - | Az^R^ *E. coli* J53 | - | - | 0.25 | 0.25 | 0.5 | 0.025 | 1 | 2 | 2 | 1 | 1 | 1 |
| - | - | TC-J53  (PEER 41_2022 + J53) | 1.25×10^-2^ | 16 | 4 | 2 | 2 | 16 | 32 | 16 | 32 | 16 | 32 |
| - | *S*. Typhi | - | - | 0.012 | 0.012 | 0.25 | 0.25 | 1 | 1 | 1 | 2 | 1 | 1 |
| - | - | TC-*S*. Typhi (PEER 41_2022 + *S*. Typhi) | 2.23 ×10^-2^ | 4 | 4 | 1 | 2 | 8 | 32 | 16 | 32 | 16 | 16 |
| - | *Klebsiella pneumoniae* | - | - | 0.25 | 0.25 | 0.5 | 0.5 | 1 | 2 | 2 | 4 | 2 | 1 |
| - | *-* | TC- *Kpn*  (PEER 41_2022 + *Kpn*) | 2.09×10^-2^ | 4 | 4 | 4 | 2 | 8 | 32 | 16 | 64 | 32 | 32 |
| - | *E.coli* | - | - | 0.5 | 0.25 | 0.5 | 0.5 | 1 | 1 | 1 | 1 | 0.5 | 1 |
| - | *-* | TC-*E. coli*  (PEER 41_2022 + *E. coli*) | 9.3×10^-3^ | 16 | 8 | 4 | 2 | 8 | 32 | 16 | 64 | 16 | 32 |
| PEER 926_2022 | *-* | - | - | 256 | 64 | 64 | 64 | 128 | 256 | 128 | 512 | 128 | 128 |
|  | *-* | TC-J53  (PEER 926_2022 + J53) | 1.85×10^-2^ | 4 | 8 | 4 | 2 | 8 | 32 | 16 | 16 | 32 | 32 |
|  | *-* | TC-*S*. Typhi (PEER 926_2022 + *S*. Typhi) | 3.2×10^-2^ | 4 | 4 | 2 | 1 | 8 | 16 | 8 | 8 | 16 | 16 |
|  | *-* | TC- *Kpn*  (PEER 926_2022 + *Kpn*) | 2.3×10^-2^ | 16 | 8 | 4 | 2 | 8 | 32 | 16 | 16 | 32 | 32 |
|  | *-* | TC- *E. coli*  (PEER 926_2022 + *E. coli*) | 22.6×10^-3^ | 8 | 8 | 4 | 2 | 8 | 32 | 16 | 16 | 16 | 32 |
| PEER 314_2023 | *-* | - | - | 128 | 64 | 64 | 256 | 128 | 256 | 128 | 512 | 128 | 128 |
| *-* | - | TC-J53  (PEER 314_2023 + J53) | 7.5×10^-3^ | 4 | 8 | 4 | 2 | 8 | 32 | 16 | 16 | 16 | 32 |
| *-* | - | TC-*S*. Typhi (PEER 314_2023 + *S*. Typhi) | 1.6×10^-2^ | 4 | 4 | 2 | 2 | 8 | 16 | 8 | 8 | 16 | 16 |
| *-* | - | TC- *Kpn*  (PEER 314_2023 + *Kpn*) | 7×10^-3^ | 16 | 8 | 8 | 2 | 8 | 32 | 16 | 16 | 16 | 32 |
| *-* | - | TC-*E. coli*  (PEER 314_2023 + *E. coli*) | 2.5×10^-2^ | 16 | 8 | 4 | 2 | 8 | 16 | 16 | 16 | 32 | 32 |

*Frequency of Transfer is calculated as number of transconjugants per recipient cell
Abbreviations - MEM - Meropenem; DOR- Doripenem; ERT - Ertapenem; COL - Colistin; CFP - Cefepime; CAZ - Ceftazidime; AZT - Aztreonam; CAT - Chloramphenicol; TET - Tetracycline; GMN - Gentamicin. Az^R –^ Sodium Azide Resistant; *Kpn – Klebsiella pneumoniae;* TC - Transconjugant
