# Supplementary material for: Emergence of concurrently transmissible mcr-9 and carbapenemase genes in bloodborne colistin-resistant Enterobacter cloacae complex isolated from ICU patients in Kolkata, India
Source: Microbiol Spectr. 2025 Feb 6;13(3):e01542-24. doi: 10.1128/spectrum.01542-24 (PMC11878022; doi:10.1128/spectrum.01542-24)
Supplement: Table S3 — Assembly metrics, biosample, and accession numbers of the CR-ECC study isolates. [file spectrum.01542-24-s0003.doc]

**Table S3. Assembly Metrics, Biosample and Accession Numbers of the CR-ECC study isolates**

| **Sample No.** | | **N50** | **L50** | **Number of Contigs** | | **N90** | **L90** | **Biosample** | **Accession No** |
| --- | --- | --- | --- | --- | --- | --- | --- | --- | --- |
| PEER350_2022 | 151,795 | | 10 | 151 | 26,439 | | 38 | SAMN34258382 | JASKNB000000000 |
| PEER374_2022 | 127,727 | | 14 | 182 | 24,373 | | 46 | SAMN36266790 | [JAUISY000000000](https://www.ncbi.nlm.nih.gov/nuccore/JAUISY000000000) |
| PEER926_2022 | 111,857 | | 16 | 145 | 26,234 | | 47 | SAMN35847867 | [JAUDFE000000000](https://www.ncbi.nlm.nih.gov/nuccore/JAUDFE000000000) |
| PEER 41_2022 | 178,986 | | 8 | 113 | 57,632 | | 26 | SAMN34258163 | JASDEZ000000000 |
| PEER314_2023 | 146,613 | | 14 | 284 | 17,457 | | 51 | SAMN39254952 | [JAYXUJ000000000](https://www.ncbi.nlm.nih.gov/nuccore/JAYXUJ000000000) |
| PEER401_2023 | 235,994 | | 7 | 91 | 52,546 | | 27 | SAMN40044731 | [JBBDHK000000000](https://www.ncbi.nlm.nih.gov/nuccore/JBBDHK000000000) |
| MDCL47_2023 | 101,258 | | 15 | 172 | 26,540 | | 51 | SAMN41024907 | JBCHJP000000000 |
| PEER455_2023 | 176,440 | | 9 | 138 | 47,517 | | 30 | SAMN41025964 | JBCHJQ000000000 |
| PEER137_2023 | 241,875 | | 7 | 163 | 47,942 | | 21 | SAMN37394856 | JAVSPR000000000 |
| PEER402_2023 | 202,968 | | 9 | 143 | 44,384 | | 30 | SAMN40074512 | [JBBDHL000000000](https://www.ncbi.nlm.nih.gov/nuccore/JBBDHL000000000) |
| MDCL28_2023 | 145,919 | | 11 | 112 | 38,712 | | 33 | **SAMN39206197** | [JAYKGI000000000](https://www.ncbi.nlm.nih.gov/nuccore/JAYKGI000000000) |
| MDCL27_2023 | 174,312 | | 9 | 123 | 54,051 | | 29 | **SAMN39206196** | [JAYKGH000000000](https://www.ncbi.nlm.nih.gov/nuccore/JAYKGH000000000) |
| MDCL26_2023 | 233,417 | | 7 | 121 | 56,157 | | 26 | **SAMN39206030** | [JAYKGG000000000](https://www.ncbi.nlm.nih.gov/nuccore/JAYKGG000000000) |
| PEER 36_2023 | 236,151 | | 7 | 162 | 68,536 | | 22 | **SAMN37518442** | JAWDEZ000000000 |
| CNCI 61_2023 | 239,652 | | 7 | 215 | 70,037 | | 22 | **SAMN39083653** | [JAYJKV000000000](https://www.ncbi.nlm.nih.gov/nuccore/JAYJKV000000000) |
| PEER149_2023 | 36,014 | | 38 | 443 | 8,642 | | 144 | **SAMN40211085** | [JBBEFW000000000](https://www.ncbi.nlm.nih.gov/nuccore/JBBEFW000000000) |
| CNCI 40_2023 | 197,925 | | 9 | 120 | 34,202 | | 30 | **SAMN39082874** | [JAYJKU000000000](https://www.ncbi.nlm.nih.gov/nuccore/JAYJKU000000000) |
| CNCI 55_2023 | 124,511 | | 13 | 107 | 35,383 | | 38 | **SAMN39052690** | [JAYERQ000000000](https://www.ncbi.nlm.nih.gov/nuccore/JAYERQ000000000) |
| PEER 38_2023 | 174,685 | | 8 | 88 | 66,823 | | 24 | **SAMN40646318** | [JBBWCD000000000](https://www.ncbi.nlm.nih.gov/nuccore/JBBWCD000000000) |
| PEER315_2023 | 122,174 | | 10 | 150 | 29,102 | | 37 | **SAMN39283064** | [JAYMYP000000000](https://www.ncbi.nlm.nih.gov/nuccore/JAYMYP000000000) |
| PEER306_2023 | 144,887 | | 11 | 220 | 27,188 | | 44 | **SAMN39254926** | [JAYXUI000000000](https://www.ncbi.nlm.nih.gov/nuccore/JAYXUI000000000) |
| PEER 62_2023 | 140,969 | | 12 | 128 | 33,347 | | 36 | **SAMN37403478** | JAVSPU000000000 |
| PEER205_2023 | 184,646 | | 8 | 146 | 30,432 | | 32 | **SAMN37401045** | JAVSPT000000000 |
| PEER150_2023 | 252,283 | | 6 | 81 | 47,942 | | 22 | **SAMN37397188** | JAVSPS000000000 |
| PEER400_2023 | 260,820 | | 6 | 94 | 49,676 | | 23 | **SAMN40078170** | [JBBDHN000000000](https://www.ncbi.nlm.nih.gov/nuccore/JBBDHN000000000) |
| CNCI 82_2023 | 301,828 | | 6 | 90 | 63,264 | | 22 | **SAMN40077912** | [JBBDHM000000000](https://www.ncbi.nlm.nih.gov/nuccore/JBBDHM000000000) |
